# The Synthesis and Biological Activity of Series of New Polyfunctional Pyrroles with the Potential to Modulate Neuronal Viability

**DOI:** 10.1007/s12013-026-02090-4

**Published:** 2026-06-10

**Authors:** Joumaa Merza, Mouhamed Alsaqati, Corinne Wills, Paul G. Waddell, Mark Ashton

**Affiliations:** 1 Faculty of Sciences, Department of Chemistry, Homs University, Homs, Syria; 2 School of Pharmacy, Faculty of Medical Sciences, Newcastle University, Newcastle-upon-Tyne, NE1 7RU UK; 3 Translational and Clinical Research Institute, Faculty of Medical Sciences, Newcastle University, Newcastle-upon-Tyne, NE1 7RU UK; 4 School of Natural and Environmental Sciences, Faculty of Science and Engineering, Newcastle University, Newcastle-upon-Tyne, NE1 7RU UK; 5 School of Natural and Environmental Sciences, Newcastle University, Newcastle-upon-Tyne, NE1 7RU UK

**Keywords:** Pyrroles, Vilsmeier reaction, substitution; Meldrum’s acid, Apoptosis, Neurotoxicity, Neurodegenerative disorders

## Abstract

The polyfunctional pyrrole 3,5-dichloro-1*H*-pyrrole-2,4-dicarbaldehyde (**2**) was reacted with malononitrile and Meldrum’s acid in a regioselective manner to yield derivatives (**4**, **5**, **6**, **8**, **9**, and **10**). Compound **8** was isolated and its structure was characterised by crystallography and 1 and 2D-^1^H, ^13^C-NMR spectroscopy. The biological effects of these derivatives were evaluated on differentiated SH-SY5Y human neuroblastoma cells, under control conditions and following Aβ exposure as an in vitro model of Alzheimer’s disease. MTT assays were performed to assess neuronal viability, and Annexin V staining was used to quantify apoptosis. Compounds **2** and **8** significantly increased cell viability in control differentiated neurons, suggesting a neurotrophic or protective effect. However, in Aβ-treated cells, all tested compounds, namely **5**, **6**, **8** and **2**, reduced viability, indicating a possible interaction between our derivatives and Aβ-induced neurotoxicity. These findings reveal that certain polyfunctional pyrrole derivatives can differentially modulate neuronal survival depending on the cellular environment. This suggests that the neuronal cell death induced by Aβ in Alzheimer’s patients can be enhanced in the presence of those compounds. Since pyrrole scaffolds are present both in biologically essential molecules and in various environmental compounds, this dual action suggests their potential relevance in understanding the mechanisms of neurodegeneration and highlights the need for further mechanistic and translational studies to explore their impact on neurodegenerative disorders, including Alzheimer’s.

**Supplementary Information:**

The online version contains supplementary material available at https://doi.org/10.1007/s12013-026-02090-4.

## Introduction

Several degenerative neurological conditions, such as Parkinson’s [1] and Alzheimer’s [2] diseases, are known to have pathophysiology where neuronal stress is a component. Providing strategies to limit the oxidative stress caused by reactive oxygen species (ROS) and reactive nitrogen species (RNS) is therefore of interest [3], and recently a series of novel pyrrole derivatives were shown to have a protective antioxidant effect in neuronal models [4, 5].

Pyrrole is an important aromatic heterocycle that is widely distributed in both synthetic and natural products, and as such, there are many synthetic routes to access the ring system [6]. 

The Vilsmeier-Haack-Arnold reaction [7] (often abbreviated to the Vilsmeier reaction), which was originally reported in the context of aromatic and heteroaromatic formylation, has been shown over time to be extremely versatile; its use has been expanded to include over 50 different functional group transformations and a range of methods for preparing heterocyclic ring systems [8, 9]. As part of an ongoing research programme, we investigated the impact of tetra-substituted pyrrole compounds and had previously reported on the use of *N*-acetyl glycine **1** with phosphoryl chloride to generate 3,5-dichloro-1*H*-pyrrole-2,4-dicarboxaldehyde **2** [10], which could then be reacted in both a chemo- and regioselective manner [11]. Within **2** there are a range of electrophilic centres that should allow for the selective synthesis of a range of substituted pyrroles.

Our findings revealed that several of these substituted pyrroles exhibited neuroprotective activity in healthy neuronal cultures, which is consistent with previous reports demonstrating that certain pyrrole derivatives possess antioxidant and neuroprotective properties [12]. However, under Alzheimer’s disease (AD) modelling conditions, characterised by β-amyloid (Aβ) toxicity, some derivatives exacerbated neuronal damage. This suggests that the biological activity of these pyrrole analogues is highly context-dependent, protective under physiological conditions but potentially deleterious in neurodegenerative environments. Such dual behaviour highlights the importance of structural and mechanistic optimisation in designing pyrrole-based therapeutics for neurodegenerative disorders.

## Method and Materials

All reagents and solvents were obtained from Sigma-Aldrich (St. Louis, MO), Fisher Scientific, (Loughborough, UK), Thermo Fisher Scientific (Waltham, MA USA), VWR International (Radnor, Pennsylvania, USA), Apollo Scientific (Denton, Manchester, UK), Manchester Organics Ltd (Runcorn Cheshire, UK), Tokyo Chemical Industry UK Ltd (Oxford, UK), and Goss Scientific Instruments Ltd(Gresty, UK), and used without further purification unless specified. Reactions requiring an inert atmosphere were conducted under nitrogen. Dry solvents were prepared using 3 Å molecular sieves before use. Melting points were determined on a Stuart SMP 10 apparatus. IR spectra (4000–650 cm⁻¹) were recorded neat using an Agilent Cary 630 FT-IR.

¹H NMR spectra were recorded on Bruker DRX spectrometers (300–700 MHz) with TMS as internal standard; chemical shifts (δ, ppm) were referenced to residual solvent peaks [DMSO-d₆ (2.50), MeOD-d₄ (3.31), CDCl₃ (7.26)]. Multiplicities are reported as s, d, t, q, m, or dd, coupling constants (J) in Hz. ¹³C NMR spectra were obtained on Bruker DRX-300 (75 MHz) or DRX-700 (175 MHz), referenced to solvent resonances [DMSO-d₆ (39.99), MeOD-d₄ (47.78), CDCl₃ (77.0)]. TLC was performed on silica gel 60 F₂₅₄ plates (Fluorochem) and visualised under 254 nm UV light. Solvents were evaporated under reduced pressure using a Buchi V-700 pump, V-850 controller, R-210 rotavapor, and B-491 heating bath. High-resolution mass spectra (HR-MS) were obtained by HPLC with electrospray ionisation (ESI⁻), using single or triple quadrupole mass analysis and QToF detection for accurate mass measurements. Analysis - Quadrupole Time-of-Flight (QToF). X-ray crystallography: The crystal structure was collected on a XtaLAB Synergy HyPix-Arc 100 diffractometer equipped with a fine-focus sealed X-ray tube (λ_CuKα_ = 1.54184 Å). Data were collected at 150 K using an Oxford Cryosystems CryostreamPlus open-flow N_2_ cooling device. Cell refinement, data collection and data reduction were undertaken via software CrysAlisPro [13] Analytical numeric absorption correction using a multifaceted crystal model based on expressions derived by Clark & Reid.

### Experiments

#### Synthesis of the Compounds 4, 5 and 6

3,5-dichloro-1*H*-pyrrole-2,4-dicarbaldehyde (200 mg, 1.04 mmol) was added to a solution of malononitrile (132.5 mg, 1.04 mmol) in 50 mL of distilled water. The reaction was heated at 65 °C for 1 h, cooled to room temperature and the resulting brown solid filtered under vacuum. The solid was dried in air overnight and purified by column chromatography using petroleum ether/AcOEt (80/20–50/50) to give the compounds **4**, **5** and **6**; the yield was 34% for **6**, 25% for **4** and 20% for **5**.

### Amberlyst Mediated Synthesis of 4 and 5

3,5-dichloro-1*H*-pyrrole-2,4-dicarbaldehyde (100 mg, 0.52 mmol) was added to a solution of 1 equivalent of malononitrile (34.4 mg, 0.52 mmol) in 50 mL of distilled water with Amberlyst-A-21 (10%). The reaction was heated at 65 °C for 1 h, cooled to room temperature and the product filtered under vacuum. The resulting brown precipitate was purified by column chromatography; compounds **4** and **5** were isolated in 35% and 30% yield, respectively.

### Direct Synthesis of Compound 6

3,5-dichloro-1*H*-pyrrole-2,4-dicarbaldehyde (100 mg, 0.52 mmol) was added to a solution of 2 equivalents of malononitrile (68.64 mg, 1.04 mmol) in 50 mL of distilled water. The reaction was heated at 65 °C for 1 h, cooled to room temperature, and the resulting brown solid was filtrated under vacuum. The solid was dried in air overnight and purified by column chromatography using petroleum ether/AcOEt (80/20–50/50) with a yield of 68% of **6** and < 2% of compound **5**.

## Synthesis of Compounds 8, 9 and 10

3,5-dichloro-1*H*-pyrrole-2,4-dicarbaldehyde 7 (100 mg, 0.52 mmol) was added to a solution of Meldrum acid (74.88 mg, 0.52 mmol) in 40 mL of distilled water. The reaction was heated at 75 °C for 1 h, cooled to room temperature and the product filtered under vacuum. The yellow precipitate (crude weight was 115 mg) was purified by column chromatography using petroleum ether/AcOEt (80/20–50/50) to give the compounds **8** (40% yield) and **9** (25% yield) with a trace of **10**.

### Direct Synthesis of Compound 8

3,5-dichloro-1*H*-pyrrole-2,4-dicarbaldehyde **7** (200 mg, 1.04 mmol) was added to a solution of Meldrum acid (150.7 mg, 1.04 mmol) in 40 mL of distilled water. The reaction was stirred overnight at room temperature, and the resulting yellow precipitate was filtered under vacuum. The crude sample was purified by column chromatography to give the compound **8**, which was recrystallised from acetone (with 5 drops of CHCl_3_).

### X-Ray Crystallography

Analysis - Quadrupole Time-of-Flight (QToF). X-ray crystallography: Single crystals of 8 were obtained by slow evaporation of the solvent from a mixture of acetone and 5 drops of chloroform. Crystal structure data for 8 were collected on an XtaLAB Synergy HyPix-Arc 100 diffractometer equipped with a fine-focus sealed X-ray tube (λ CuKα = 1.54184 Å). Data were collected at 150 K using an Oxford Cryosystems CryostreamPlus open-flow N_2_ cooling device. Cell refinement, data collection and data reduction were undertaken via software CrysAlisPro [14]. Analytical numeric absorption correction using a multifaceted crystal model based on expressions [15].

All structures were solved using XT [16] and refined by XL [17], using the Olex2 interface [18]. All non-hydrogen atoms were refined anisotropically, and hydrogen atoms were positioned with idealised geometry, except for those bound to heteroatoms, the positions of which were located using peaks in the Fourier difference map. The displacement parameters of the hydrogen atoms were constrained using a riding model with U_(H)_ set to be an appropriate multiple of the U_eq_ value of the parent atom.

The crystal for which data were collected was a two-component inversion twin. The asymmetric unit of the structure comprises two crystallographically-independent molecules. The salient difference between the two independents is the orientation of the aldehyde group, though some more subtle conformational variations are apparent. Both molecules exhibit an intramolecular hydrogen that forms a seven-membered ring motif with the graph set R(7) [19].

### Protein–Protein Docking Analysis and Aβ–Compounds Binding Energies

Computational studies were carried out using BIOVIA Materials Studio 2023, Accelrys Inc. (San Diego, CA, USA). All the generated assemblies were energy minimised, and their geometry was optimised using the “Forcite module” with smart algorithms. The forcefield was set to “universal”, and charges were assigned using “use current”, and the quality “ultra fine”. Ligand and receptors were excited using https://gramm.compbio.ku.edu/ using free docking methodology.

### SH-SY5Y Cell Culture and Cholinergic Neuronal Differentiation

**Fig. 1:**
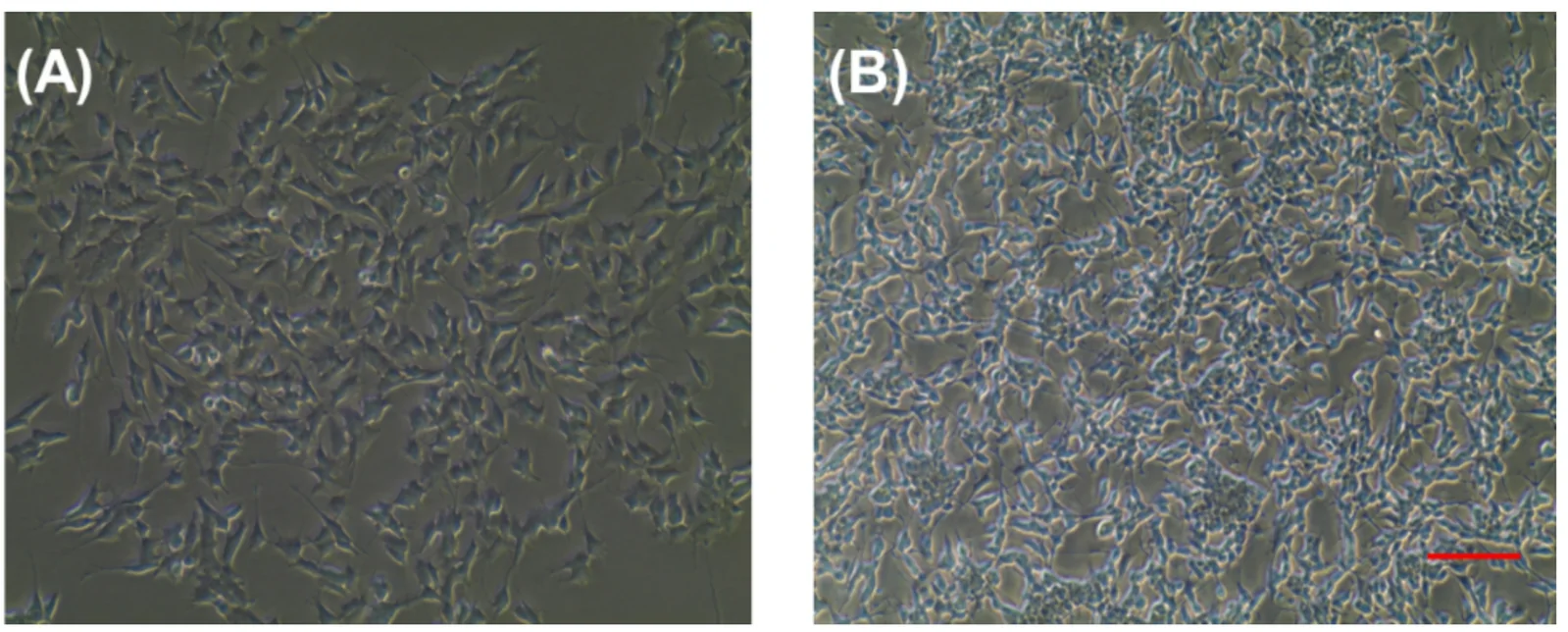
Differentiation properties of retinoic acid. **A** Undifferentiated SH-SH5Y cells. **B** Differentiated SH-SY5Y cells cultured with RA (10 µM) for 7 days. Scale bar = 50 μm

The neuroblastoma cell line (SH-SY5Y) used here was obtained from Prof Emma Kidd (Cardiff University). All cell culture components were purchased from Thermo Fisher Scientific (Waltham, MA USA) unless otherwise stated. Cells were maintained in growth medium containing Dulbecco’s Modified Eagle Medium/F-12 Nutrient Mixture (DMEM/F-12) supplemented with 10% FBS, 0.1 mM non-essential amino acids, 2 mM L-glutamine, and 10,000 U/mL penicillin and 10,000 g/mL streptomycin. For cholinergic neuronal differentiation, 96-well plates were coated with 10 ug/mL Poly-D-lysine for 2 h. Then SH-SY5Y cells were seeded at 2.5 k per cm^2^ density on Poly-D-lysine, in the growth medium and incubated at 37 °C in a humidified atmosphere containing 5% CO₂ and 95% air. On the following day, the growth medium was switched to the differentiation medium containing Dulbecco’s Modified Eagle Medium/F-12 Nutrient Mixture (DMEM/F-12) (Gibco) supplemented with 2% FBS, non-essential amino acids (Gibco), 2 mM L-glutamine, 1% penicillin/streptomycin and 10 µM retinoic acid (RA) (Tocris Bioscience, Bristol, UK). Cells were left in the differentiation medium for 7 days. At this stage, the cells expressed neurite extensions, which indicates a more developed neuronal structure, such as outgrowth of neurites and reduced cell division; A shows the undifferentiated cells, and B shows differentiated cells with short neurites, consistent with previously reported maturation patterns of SH-SY5Y cells [20] (Fig. 1).

### MTT Assay and Cell Viability

Cell viability, reflecting mitochondrial metabolic activity, following exposure to compounds **5**, **6**, **8** and **2** was assessed using a quantitative colorimetric MTT assay (ab211091, Abcam, Cambridge, UK), as previously described by Denizot and Lang (1986) [21]. Briefly, 50 µL of serum-free medium and 50 µL of MTT reagent were added to each well, including vehicle control (DMSO) and blank wells. Plates were incubated at 37 °C in a humidified atmosphere containing 5% CO₂ and 95% air for 3 h. Following incubation, 150 µL of MTT solvent was added to each well. The plates were then wrapped in foil and agitated on an orbital shaker for 15 min to ensure complete dissolution of the formazan crystals.

Metabolically active cells reduce the yellow MTT tetrazolium salt to an insoluble purple formazan product, which was subsequently solubilised in DMSO. Absorbance was measured at 590 nm using a plate reader. For each condition, readings were averaged and corrected by subtracting the blank values. Cell viability was expressed as a percentage relative to vehicle control cells treated with DMSO, which were defined as 100% viability.

### Apoptosis Detection Assay

Differentiated SH-SY5Y cells were incubated in the presence or absence of **5**, **6**, **8** and **2** or vehicle for 48 h. A staining mixture containing 1:100 Annexin V, and 1:1000 Hoechst (Thermo Fisher Scientific, Waltham, MA USA) was prepared in binding assay buffer following the manufacturer’s instructions (BioLegend, San Diego, CA). Cells were washed with Hanks’ Balanced Salt Solution (HBSS) (Thermo Fisher Scientific, Waltham, MA USA) and incubated with the mixture at 37 °C in a humidified atmosphere containing 5% CO₂ and 95% air for approximately 30–60 min. Post incubation, the cells were gently washed with fresh Annexin binding buffer and placed on ice and imaged using Zeiss LSM800 confocal microscopy. Images were captured from five fields per well, and the number of apoptotic cells in each field was counted. These counts were then averaged to obtain a value for each well. The percentage of apoptotic cells for each condition was calculated relative to the total number of live cells, which were identified using Hoechst nuclear staining.

### Preparation of Aβ (1–42)

Aβ (β-Amyloid 1–42) was purchased from Eurogentec (Seraing, Belgium). A stock solution was prepared by dissolving 1 mg of Aβ (1–42) peptide in 100 µL of 10 mM NaOH. The solution was then diluted to a final volume of 0.5 mL with Milli-Q water, followed by the addition of 0.5 mL of phosphate buffer (pH 7.4). The resulting mixture was centrifuged at 16,000 × g for 10 min and used to prepare a final stock concentration of 1 mg/mL.

### Statistical Analysis

Prism 10.0 (GraphPad Software) was used for the statistical analysis. Data shown are the mean ± s.e.m. with *p* < 0.05 considered statistically significant. Group differences were analysed with one-way analysis of variance (ANOVA) followed by Dunnett’s multiple comparisons test.

## Results and Discussion

### Compounds Synthesis

**Scheme 1:**
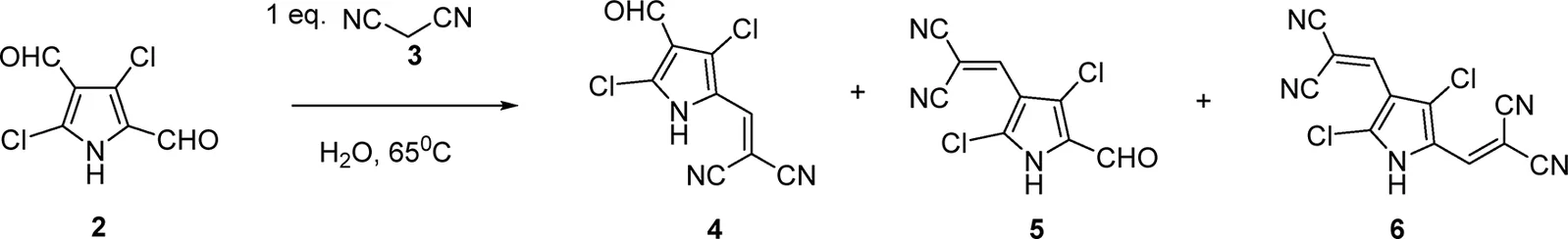
Reaction of 3,5-dichloro-1H-pyrrole-2,4-dicarbaldehyde 2 with one equivalent of malononitrile 3 in distilled water at 65 °C, affording compounds 4, 5 and 6 after purification by column chromatography. Isolated yields: 25% for 4, 20% for 5, and 34% for 6.

Starting with 3,5-dichloro-1*H*-pyrrole-2,4-dicarboxaldehyde **2** and following on from previous work, we first explored a Knovenagel condensation [22] with malononitrile **3**; conditions included both 1 equivalent of malononitrile and 2 equivalents and also the use of Amberlyst-21 (10 mol %) as catalyst (Scheme 1). When employing 1 equivalent of malononitrile in water at 65 °C, we observed a slight regioselectivity in favour of the 2-formyl group **4** compared to the 4-formyl group **5**, yielding 34% versus 20%, respectively, and a proportion of the di-substituted compound (**6**, 44% yield). Two equivalents of malononitrile gave **6** in 65% yield with only traces of **4** and **5**. When employing the eco-friendly Amberylst-21 (10 mol %) as catalyst, with 1 equivalent of malononitrile at room temperature, the relative composition of **4**:**5**:**6** was 45%, 25%, and trace, respectively.

The versatility of Meldrum’s acid [23] and its derivatives in organic synthesis is well known, and indeed, the Knoevenagel condensation of Meldrum’s acid **7** with aldehydes leads to alkylidene derivatives which can be further reacted. The synthesis of alkylidene derivatives from the corresponding aldehyde and Meldrum’s acid can be achieved under a variety of conditions, but we focussed our attention on the reaction of **2** with Meldrum’s acid in water *via* a modification of the method of Bigi [24].

Addition of 2 equivalents of Meldrum’s acid (7) to a suspension of compound **2** in water at room temperature afforded compounds **8** and **9** in 40% and 10% yield, respectively, with only trace amounts of compound 10 observed.

Repeating the reaction at 75 °C resulted in a slight increase in the yield of compound **9** (25%), while the yields of compounds 8 and 10 remained largely unchanged.

However, when the reaction was carried out at 75 °C between compound 7 (2 equivalents) and a suspension of compound 2 in water, using Amberlyst-21 (10 mol%) as a heterogeneous catalyst, compound **8** was obtained in 60% yield, while compound **9** was formed in only 5% yield.

**Scheme 2:**
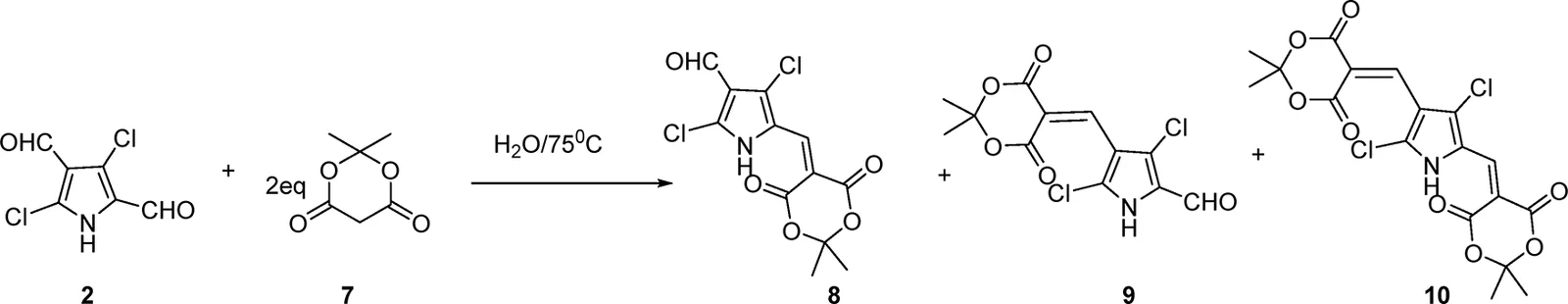
Reaction of 3,5-dichloro-1H-pyrrole-2,4-dicarbaldehyde 2 with Meldrum’s acid 7 in distilled water at 75 °C, yielding compounds 8 and 9 after purification by column chromatography, together with a trace amount of compound 10. Isolated yields: 40% for 8 and 25% for 9.

**Fig. 2:**
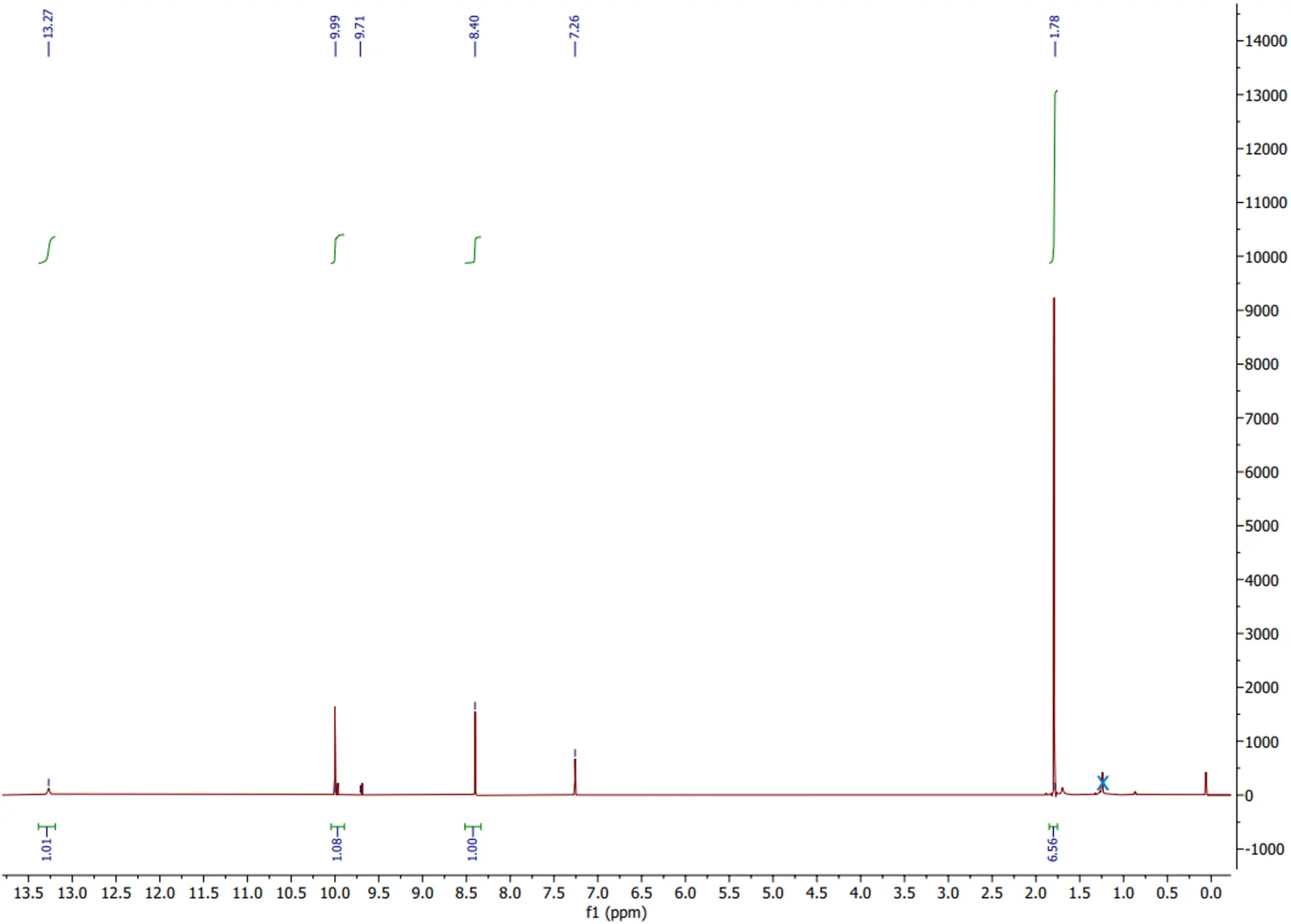
^1^H NMR spectrum for 8

**Fig. 3:**
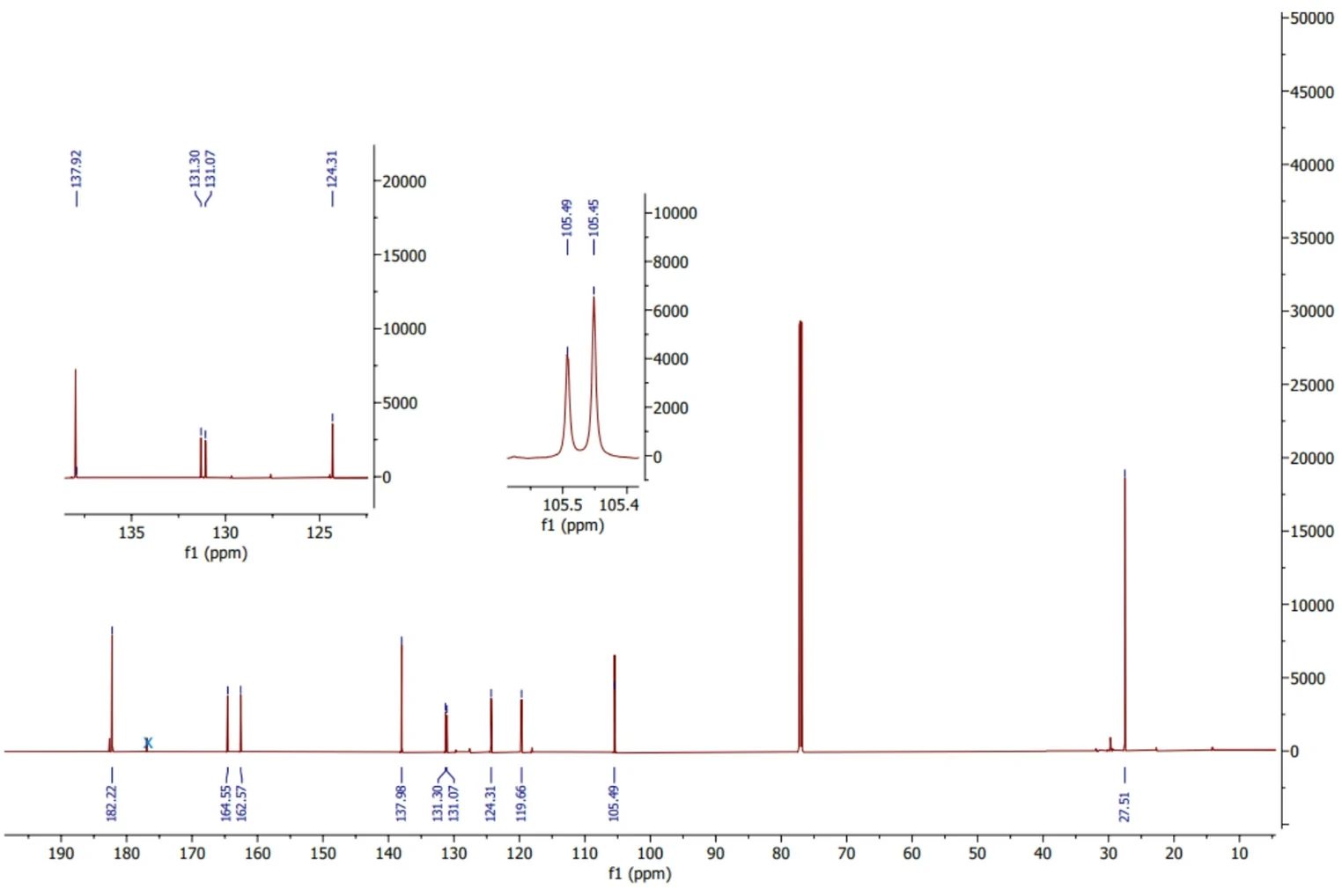
^13^C NMR spectrum for 8

**Fig. 4:**
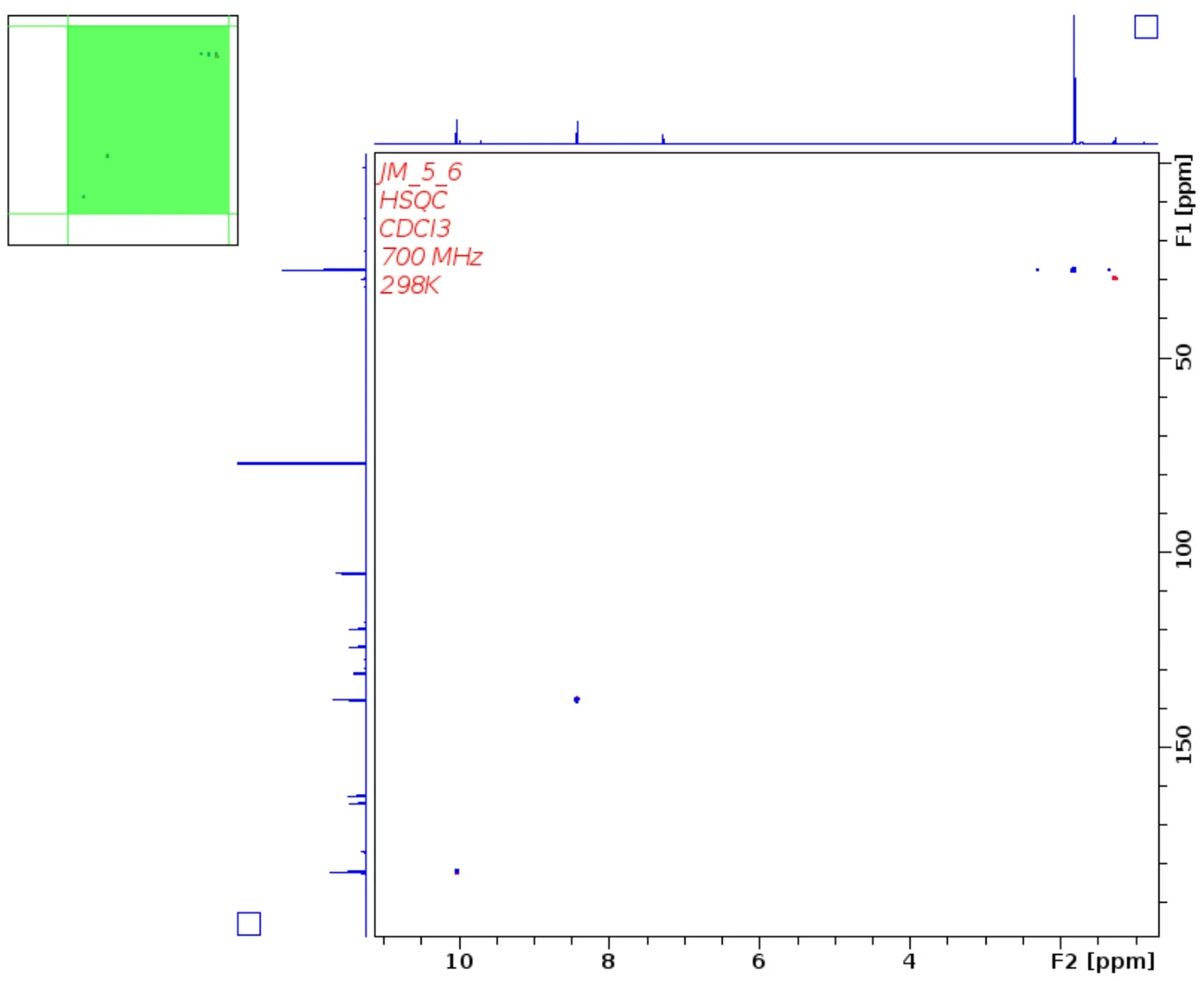
HSQC spectrum for 8 (700 MHz)

**Fig. 5:**
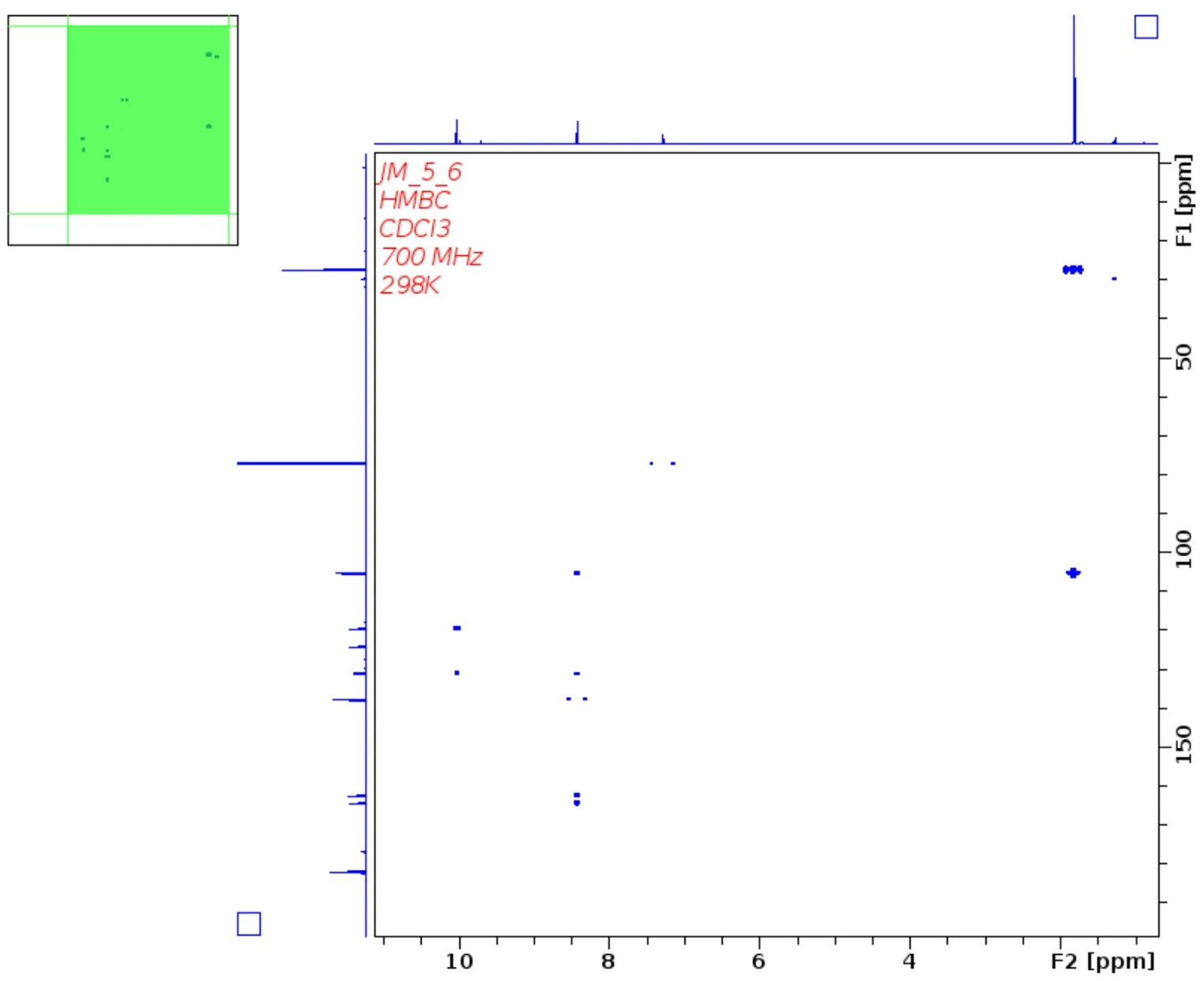
HMBC spectrum for 8 (700 MHz)

**Fig. 6:**
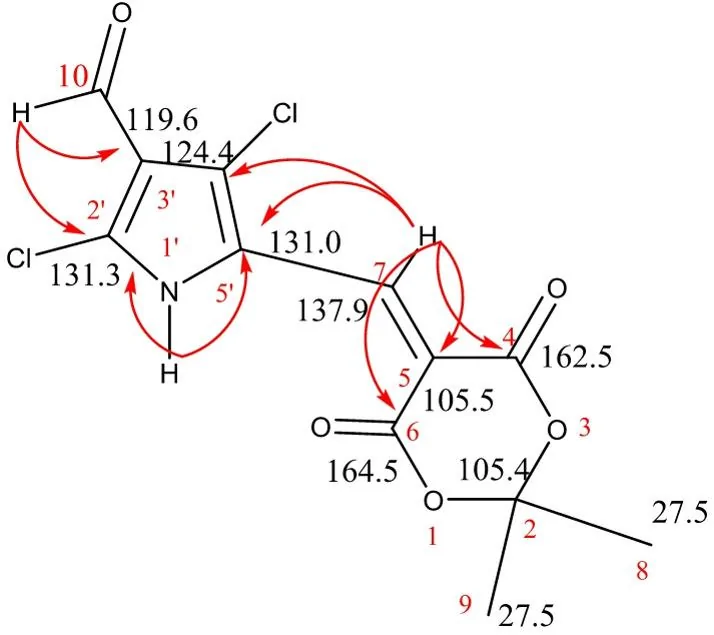
assignment of ^13^C and correlation H-C based on HSQC and HMBC experiments

**Fig. 7:**
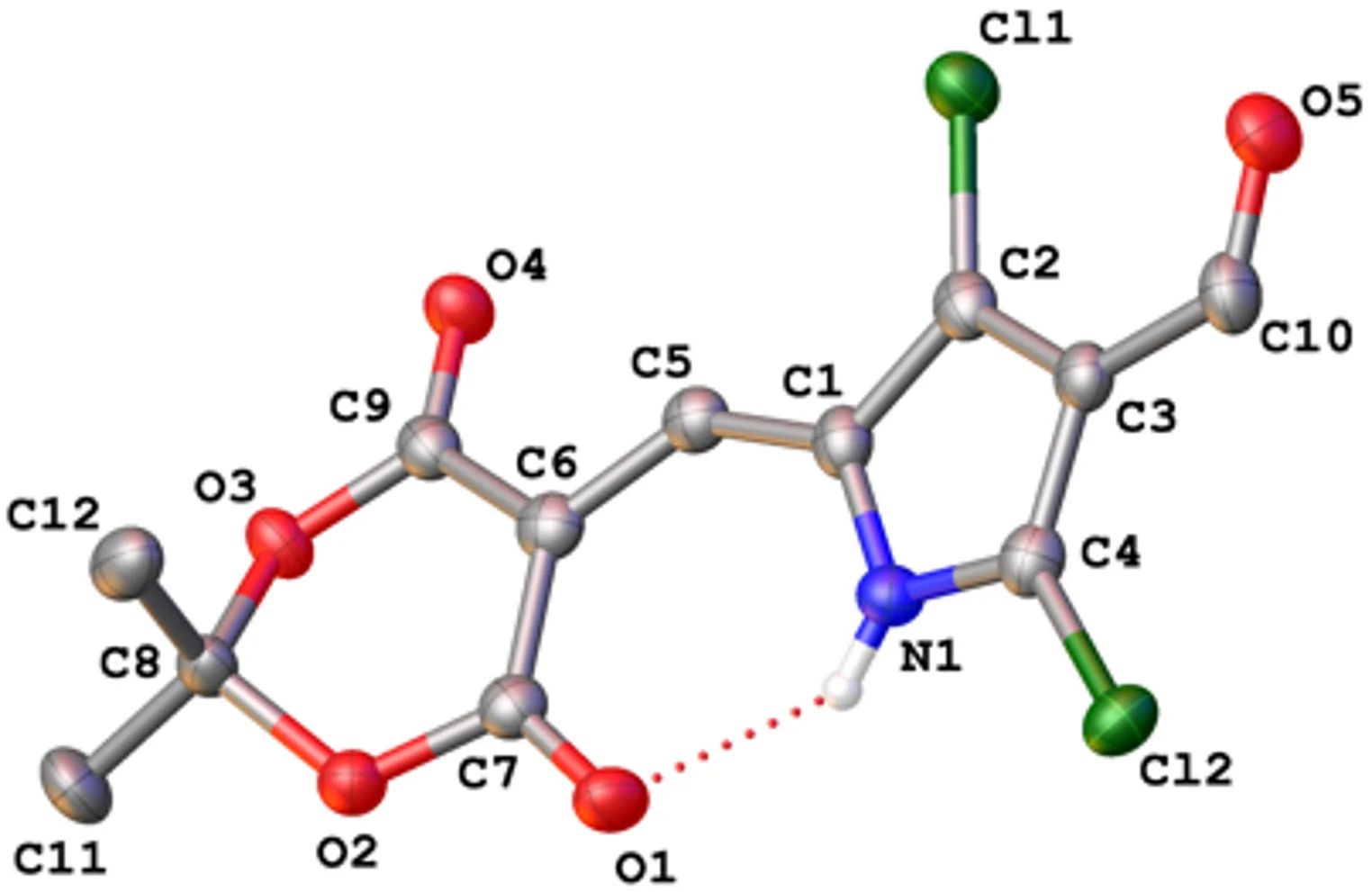
The crystal structure of 2,4-dichloro-5-[(2,2-dimethyl-4,6-dioxo-1,3-dioxan-5-ylidene) methyl]-1*H*-pyrrole-3-carbaldehyde 8 with ellipsoids drawn at the 50% probability level. Only one of the two independent molecules in the asymmetric unit is shown, and hydrogen atoms bonded to carbon atoms have been omitted for clarity

The products obtained from the reaction of **2** with **7** were highly coloured crystalline solids after purification and the regioselectivity was assigned as 2,4-dichloro-5-[(2,2-dimethyl-4,6-dioxo-1,3-dioxan-5-yldiene)methyl]-1*H*-pyrrole-3-carboxaldehyde **8** and 3,5-dichloro-4-[(2,2-dimethyl-4,6-dioxo—1,3-dioxan-5-ylidene)methyl]-1*H*-pyrrole-2-carbaldehyde **9** (Scheme 2). Analysis of the ^1^H NMR spectra revealed that both **8** and **9** were adducts that were formed from a 1:1 molar ratio of reactants. The regiochemistry of each adduct was determined using ^1^H and ^13^C NMR spectroscopy (Figs. 2 and 3), together with 2D NMR experiments, including HSQC (Fig. 4) and HMBC (Fig. 5). The absolute configuration and complete atomic assignment of compound 8 were further confirmed by single-crystal X-ray diffraction analysis (Figs. 6 and 7). All chemical shifts and corresponding H–C correlations are summarised in Table 1. The crystal structure and refinement details are summarised in Table 2. The remaining compounds (4, 5, 6, 7, and 9) were characterised using the same methodology (Supplement Information).

**Table 1:** assignment of ^1^H and ^13^C signals in regioisomer 8

*N* ^0^	δ_H_	δ_C_	*N* ^0^	δ_H_	δ_C_	HSQC	HMBC
**1**	13.27	-	**7**	8.40 (1 H, s)	137.9	137.9	162.5 (C-6), 164.5 (C-4), 105.5 (C-5), 131.0 (C-5’), 124.4 (C-4’)
**2**	-	105.4	**1’**	13.9	-	-	-
**3**	-	-	**2’**	-	131.3	-	-
**4**	-	162.5	**3’**	-	119.6	-	-
**5**	-	105.4	**4’**	-	124.4	-	-
**6**	-	164.5	**5’**	-	131.0	-	-
**8**,** 9**	1.78 (6 H, s)	27.5	**10 (β-CHO)**	9.99 (1 H, s)	182.2	-	131.3 (C-2’), 119.6 (C-3’)

**Table 2:** Crystal data and structure refinement for 8

Empirical formula	C_12_H_9_Cl_2_NO_5_
Formula weight	318.10
Temperature/K	150.0(2)
Crystal system	monoclinic
Space group	P2_1_
a/Å	7.2306(2)
b/Å	22.5895(5)
c/Å	8.4353(2)
α/°	90
β/°	109.136(3)
γ/°	90
Volume/Å^3^	1301.65(6)
Z	4
ρ_calc_g/cm^3^	1.623
µ/mm^− 1^	4.689
F(000)	648.0
Crystal size/mm^3^	0.2 × 0.1 × 0.03
Radiation	CuKα (λ = 1.54184)
2Θ range for data collection/°	7.828 to 154.462
Index ranges	-8 ≤ h ≤ 9, -27 ≤ k ≤ 27, -10 ≤ l ≤ 9
Reflections collected	18,643
Independent reflections	5143 [R_int_ = 0.0314, R_sigma_ = 0.0269]
Data/restraints/parameters	5143/1/374
Goodness-of-fit on F^2^	1.028
Final R indexes [I > = 2σ (I)]	R_1_ = 0.0327, wR_2_ = 0.0841
Final R indexes [all data]	R_1_ = 0.0336, wR_2_ = 0.0850
Largest diff. peak/hole / e Å^−3^	0.89/-0.27
Flack parameter	0.310(14)

### Protein–Protein Docking Analysis and Aβ–Compounds Binding Energies

**Fig. 8:**
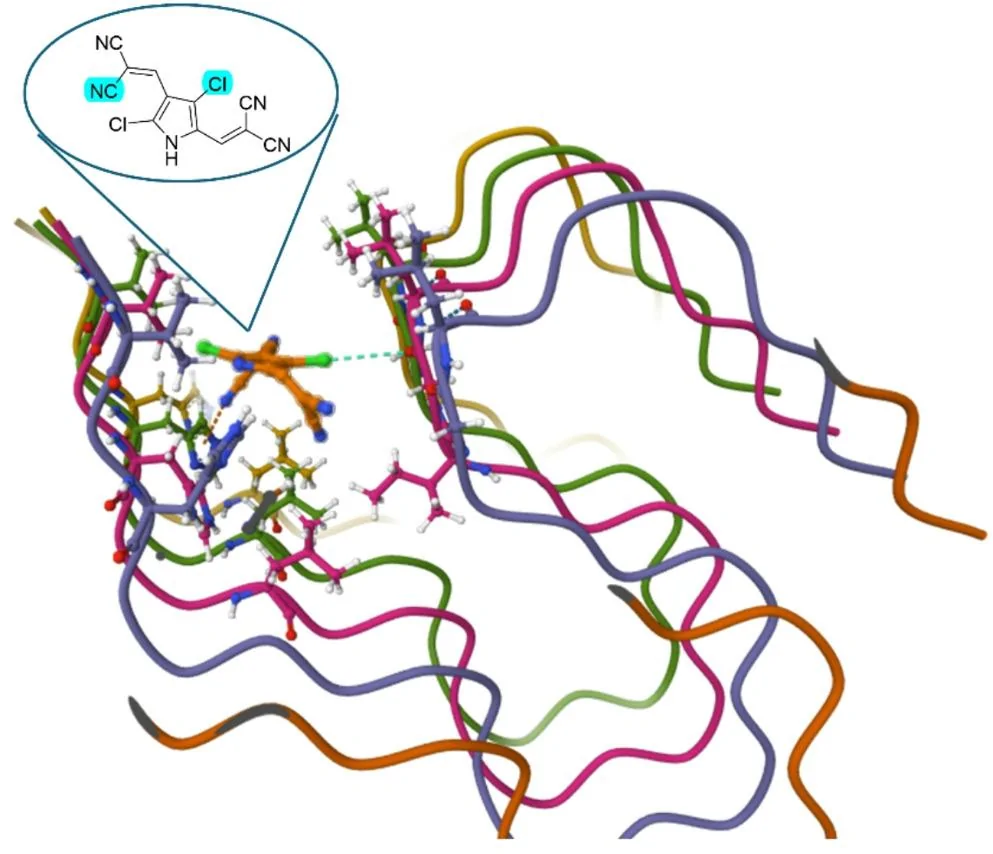
GRAMM docking of compound **6** with the Aβ complex. A close-up view highlights the predicted interactions, including a hydrogen bond between the Cl (5) substituent and the carbonyl group of Gly33 in polymer 4, as well as a hydrogen bond between the CN group and the imidazole ring of His14 in polymer 5

Molecular modelling was used to evaluate the binding affinity and interactions between our compounds and Aβ. The calculated Gibbs free binding energies indicated that compounds **8**, **6**, and **5** exhibited higher binding affinities (-131, -126, and − 101 kcal/mol, respectively) compared to compounds **2** (-76 kcal/mol, respectively), suggesting more favourable interactions with Aβ (Table 3). Furthermore, protein–ligand docking analysis using GRAMM revealed specific interaction between our compounds and Aβ complex [25]. For example, compound **6** formed a hydrogen bond between its Cl (5) substituent and the carbonyl group of Gly33 in Aβ polymer 4, while the CN group established a hydrogen bond with the imidazole ring of His14 in Aβ polymer 5 (Fig. 8).Together, these findings indicate that our compounds can interact with Aβ, which may contribute to modulation of its toxic activity.

**Table 3:** Binding energy of compound 6

Compound	Binding energy (Kcal/mol)
2	-76
5	-101
6	-126
8	-131

### Effect of the Compounds on Neuronal Viability in a Model of AD

Several studies suggested the involvement of pyrrole or nitrate groups in the regulation of neuronal cell viability [26, 27]. To assess the neurotoxic effects of our four compounds, we evaluated the viability of SH-SY5Y cells differentiated into cholinergic neurons using an MTT assay. The MTT assay is a commonly used method that offers insights into cell metabolic activity. Reduced cellular metabolic activity serves as a marker for compromised cell function.

**Fig. 9:**
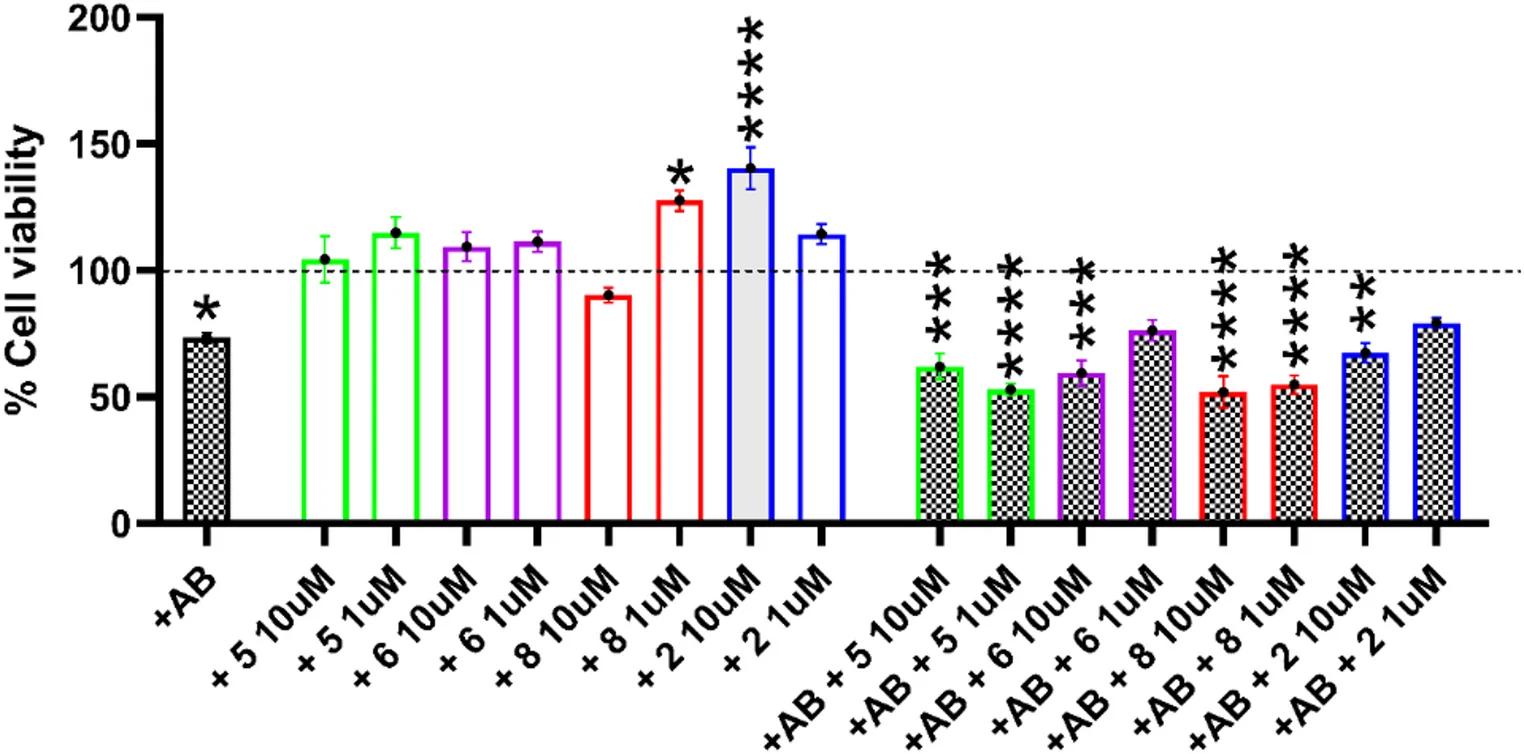
Effect of **5**,** 6**,** 8** or **2** compounds on the viability of differentiated SH-SY5Y cells following 48-hour incubation, in the absence or presence of 3 µM Aβ, as assessed by MTT assay. Data are expressed as the percentage of values in untreated control cultures, shown as a dotted line. Each value indicates a mean ± SEM (*n* ≥ 3). Data were presented as mean ± SEM and were analysed by One-way ANOVA with post hoc comparisons using Dunnett’s multiple comparisons test comparing to control cells. * *p* < 0.05, ** *p* < 0.01, *** *p* < 0.001, **** *p* < 0.0001

For this experiment, several concentrations (100 nM, 1 µM, 10 µM, and 20 µM) were initially tested in differentiated SH-SY5Y cells following 24- or 48-hour incubations to observe any potential cell death with any of the compounds. No significant cell death was observed at any concentration or time point. Based on these initial optimisations, 1 µM and 10 µM were selected for subsequent experiments, using a 48-hour incubation period to assess the effects of longer-term exposure on cell viability, both in the absence and presence of 3 µM Aβ.

to investigate the effect of these compounds on neuronal growth in healthy neuronal setups or in neuronal models of Alzheimer’s disease. Results showed that SH-SY5Y cell viability was slightly improved in the presence of **5**, **6**, **8** and **2**, but the increase in the cell viability only reached statistical significance in the presence of 1µM **8** or 10µM **2** (*P* < 0.05, *P* < 0.001, respectively). While cells incubated with 3 µM Aβ exhibited approximately 25% reduction in their viability (*P* < 0.05) (Fig. 9). Interestingly, cells co-treated with Aβ and any of the above compounds exhibited significantly greater reductions in their viability compared to those treated with Aβ alone; for example, cells co-treated with Aβ and 10 µM C8 showed approximately 50% reductions in their viability (*P* < 0.0001) (Fig. 9). These compounds may enhance neuronal viability in healthy cells by supporting cellular metabolism, promoting neurotrophic signalling, or reducing baseline oxidative stress [12, 28]. However, in diseased neurons, such as those exposed to Aβ, these compounds may exacerbate existing cellular stress or interact adversely with pathological mechanisms, resulting in toxicity. These observations suggest that our pyrrole derivatives could serve as valuable tools for modelling neurodegenerative disease in vitro and for investigating their interactions with potential therapeutic agents used to manage neurodegenerative disorders.

**Fig. 10:**
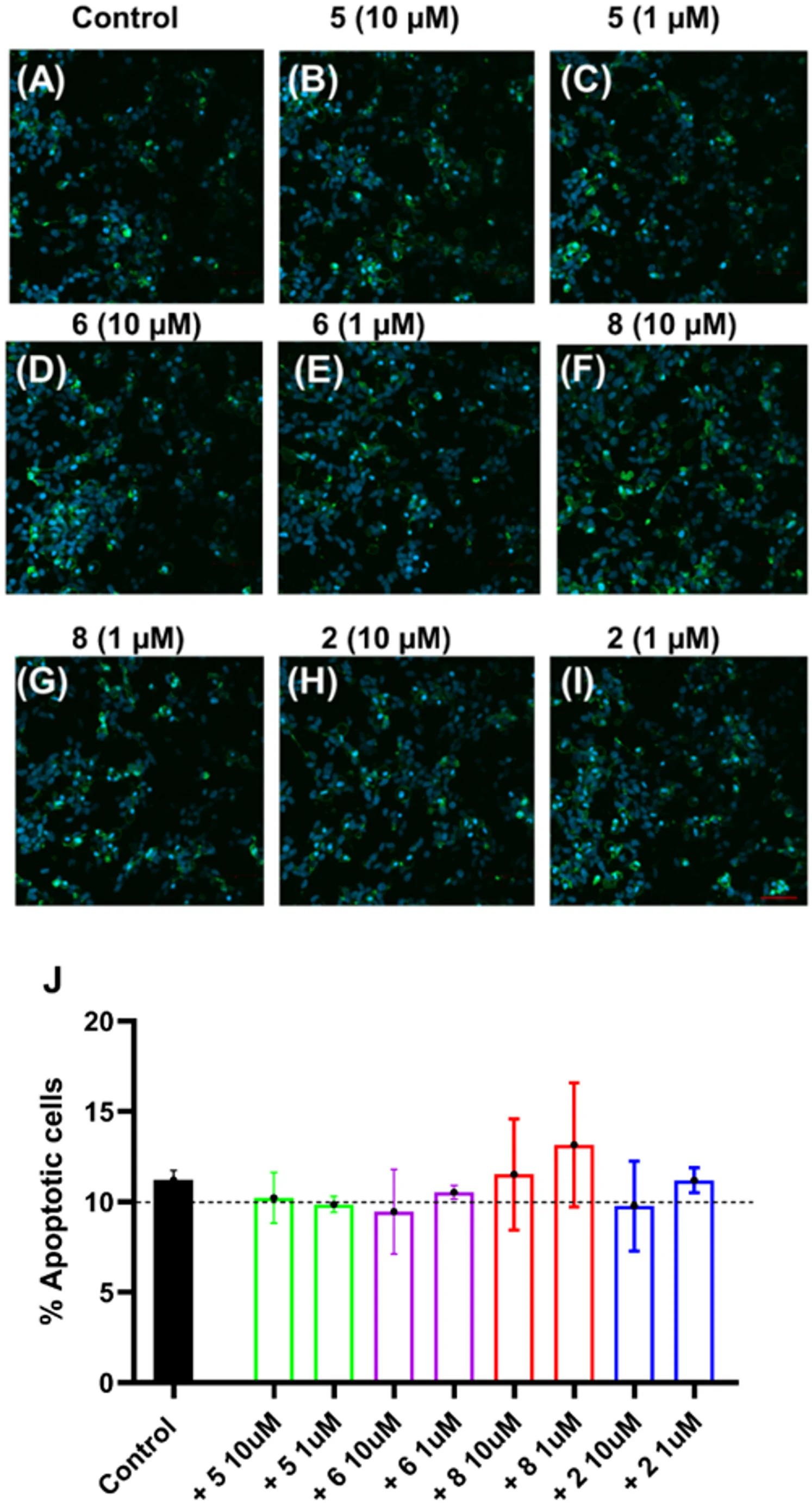
Live and apoptotic differentiated SH-SY5Y cells in the absence (A) or presence of **5** (10 µM) (B), **5** (1 µM) (C), **6** (10 µM) (D), **6** (1 µM) (E), **8** (10 µM) (F), **8** (1 µM) (G), **2** (10 µM) (H) or **2** (1 µM) (I) under a confocal microscope. Assays were done with live (Hoechst; blue) and apoptotic cells (Annexin V; green) by using a confocal microscope captured following 48 h incubation with the compounds. The scale bar was set at 50 μm. (J) Graphs show the percentage of apoptotic cells to total number of cells in vehicle control cells and cells treated with 5, 6, 8 or 2. Data were presented as mean ± SEM and were analysed by One-way ANOVA with post hoc comparisons using Dunnett’s multiple comparisons test comparing to control cells

We then examined whether any of these compounds might induce apoptosis in SH-SY5Y cells differentiated into cholinergic neurons. Following treatment of differentiated cells with **5**,** 6**,** 8** and **2** at 1 µM or 10 µM concentrations or 0.1% tween as a positive control for 48 h, cells were treated with Annexin V and Hoechst, and the fluorescent intensity was measured. Results showed that there was no significant difference in the level of the apoptotic cells between vehicle control cells and cells treated with any of the four compounds. In contrast, treatment with 0.1% Tween caused substantial cell death, resulting in an approximate 30% increase in apoptotic cells This indicates that none of the compounds would induce an apoptotic response in our neurons (Fig. 10).

These results indicate that, in the absence of Aβ, the compounds do not induce detectable apoptosis in healthy neurons. In contrast, treatment of differentiated SH-SY5Y cells with Aβ in combination with our compounds resulted in a greater reduction in cell viability compared to Aβ treatment alone. This effect was more pronounced when Aβ was incubated with compounds **6** and **8**, which is consistent with our molecular docking and protein–protein interaction analyses showing that these compounds have higher binding affinities to Aβ than compounds **2** and **4**. Previous studies have demonstrated the pyrrole-forming potential of γ-diketones, such as 2,5-hexanedione and its perdeuterated analogue, which induce neurotoxicity through the formation of pyrrole adducts on lysine residues [29]. While Aβ peptides alone do not provide the carbonyl groups required for this reaction, they can promote oxidative stress, mitochondrial dysfunction, and lysine residue modifications, which may increase the susceptibility of lysine residues to react with exogenous γ-diketones [30, 31]. Moreover, Aβ-stimulated stress can enhance protein carbonylation [31], crosslinking, and advanced glycation end-product formation processes [32], mechanistically similar to pyrrole adduct generation, potentially contributing to neurofilament or cytoskeletal injury and synergising with γ-diketone toxicity. In addition, the interaction between our compounds practically **6** and **8** and Aβ interaction may act synergistically with Aβ, contributing to the observed enhancement of toxicity, which is not observed when the compounds are administered individually.

.

## Conclusions

Six new polyfunctionalized pyrrole derivatives were synthesized by regioselective reaction between the polyfunctional pyrrole 2 and Meldrum’s acid and malononitrile in two steps, using ecofriendly conditions of water as solvent and Amberlyst A-21 as heterogenous catalyst. The single crystalline of **8** was obtained. The effects of **2**, **5**,** 6** and **8** have been evaluated on neuronal cell viability in healthy cellular models and in those of the AD model. In healthy neurons, compounds **2** and **8** improve neuronal viability, while compounds **5** and **6** show no significant effect. In contrast, in the Alzheimer’s model, all of these compounds reduce neuronal viability. Given that pyrrole compounds are found in both biologically essential molecules and various environmental substances, this study enhances our understanding of how pyrrole-related structures may influence neuronal health and toxicity. In addition, our novel derivatives could serve as useful tools for modelling AD in vitro and for evaluating their interactions with potential therapeutic agents. Further research is required to evaluate the antioxidant properties of our compounds and to elucidate the mechanisms underlying their neuroprotective effects.

## Supplementary Information

Below is the link to the electronic supplementary material.

Supplementary Material 1

## Data Availability

CCDC 2390238 contain the supplementary crystallographic data for this paper. These data can be obtained free of charge via [www.ccdc.cam.ac.uk/data\_request/cif] (http:/www.ccdc.cam.ac.uk/data_request/cif) , or by emailing [data\_request@ccdc.cam.ac.uk] (mailto: data_request@ccdc.cam.ac.uk) , or by contacting The Cambridge Crystallographic Data Centre, 12 Union Road, Cambridge CB2 1EZ, UK; fax: +44 1223 336033 Other data will be available on request. The ICC and apoptotic data are available from the corresponding author upon reasonable request.
