# Supplementary material for: The Synthesis and Biological Activity of Series of New Polyfunctional Pyrroles with the Potential to Modulate Neuronal Viability
Source: Cell Biochem Biophys. 2026 Jun 10;84(3):3817–29. doi: 10.1007/s12013-026-02090-4 (PMC13585865; doi:10.1007/s12013-026-02090-4)
Supplement: Supplementary file 1 [file 12013_2026_2090_MOESM1_ESM.docx]

**The synthesis and biological activity of series of new polyfunctional pyrroles**

**with the potential to modulate neuronal viability**

**Joumaa Merza^a,b^, Mouhamed Alsaqati^a,d*^, Corinne Wills^c^, Paul G. Waddell^e^**

**Mark Ashton^a,d*^**

^a^School of Pharmacy, Faculty of Medical Sciences, Newcastle University, Newcastle-upon-Tyne, NE1 7RU, UK

^b^ Homs University, Faculty of Sciences, Department of Chemistry, Homs, Syria

^c^ School of Natural and Environmental Sciences, Faculty of Science and Engineering, Newcastle University, Newcastle-upon-Tyne, NE1 7RU, UK

^d^Translational and Clinical Research Institute, Faculty of Medical Sciences, Newcastle University, Newcastle-upon-Tyne, NE1 7RU, UK

^e^School of Natural and Environmental Sciences, Newcastle University, Crystallography, Newcastle upon Tyne, NE1 7RU, UK

**E-mail addresses:**

Dr. Joumaa Merza, [mjoma10@yahoo.com](mailto:mjoma10@yahoo.com)

Dr. Mohamad Alsaqati, [Mouhamed Alsaqati@ newcastle.ac.uk](mailto:%20Mouhamed%20Alsaqati@ncl.ac.uk)

Dr. Corinne Wills, [corinne.wills@newcastle.ac.uk](mailto:corinne.wills@newcastle.ac.uk)

Dr. Paul Waddell, [Paul.Waddell@newcastle.ac.uk](mailto:Paul.Waddell@newcastle.ac.uk)

^*^Corresponding Author – Dr. Mark Ashton**,** [Mark.Ashton@ncl.ac.uk](mailto:Mark.Ashton@ncl.ac.uk)

^*^Corresponding Author – Dr. Mohamad Alsaqati, [Mouhamed Alsaqati@ newcastle.ac.uk](mailto:%20Mouhamed%20Alsaqati@ncl.ac.uk)

**Supporting information**

Synthesis of 3,5-dichloro-1H-pyrrole-2,4-dicarboxaldehyde

**Compound 4** (2-((3,5-dichloro-4-formyl-1H-pyrrol-2-yl)methylene)malononitrile)**:**

**MP:** decomposed >250^0^C

**IR:** 3442 cm⁻¹ (N-H), 2924.7 cm⁻¹ and 2853.17 cm⁻¹ (C-H, CHO- stretching) , 1645 cm⁻¹ (C=O), 2219 (C≡N stretch).

**^1^H-NMR** (700 MHz, CDCl_3_) δ_H_: 9.98 (1H, s, H-4), 9.98 (1H, s, H-5), 7.69 (1H, s, H-1).

**^13^C-NMR** (700 MHz, CDCl_3_) δ**_C_** : 181.6 (C-5), 140.5 (C-3), 131.8 (C-5’), 127.8 (C-3’), 122.9 (C-2’), 118.8 (C-4’), 114.5 (C-3), 112 (C-1), 75.1 (C-2),

[M-H] is at m/e (237.9581), Calculated Mass (237.9580) Formula [M-H]^-^ (C_9_H_2_Cl_2_N_3_O)

**Data for compound 4**

IR spectrum of 4

**Mass spectrum of 4**

^1^H-NMR spectrum of compound 4 (700 MHz, CDCl_3_, δ_TMS_= 0 ppm)

^13^C-NMR spectrum of compound 4 (176 MHz, CDCl_3_, δ_TMS_= 0 ppm)

Table 1: ^13^C- and ^1^H chemical shifts of compound 4

| **N** | **δ_H_** | **δ_C_** | **N** | **δ_H_** | **δ_C_** |
| --- | --- | --- | --- | --- | --- |
| **1** | - | 112.8 | **1**' | - | - |
| **2** | - | 75.1 | **2**' | - | 122.9 |
| **3** |  | 114.5 | **3**' | - | 127.8 |
| **4** | 7.69 | 140.5 | **4**' | - | 118.8 |
| **5** | 9.98 (1H, s) | 181.6 | **5'** | - | 131.8 |

**Compound 5** (2-((3,5-dichloro-4-formyl-1H-pyrrol-2-yl)methylene)malononitrile):

**MP:** decomposed >250^0^C

**IR (ATR, Methanol):** 3302.1 cm^-1^ N-H stretch, 2221 cm⁻¹ (C≡N stretch), 1647 cm⁻¹: (C=O aldehydes stretching).

**^1^H-NMR** (700 MHz, CDCl_3_): δ_H_ 10.59 (1H, bd-1’), 9.71 (1H, s, H-5), 7.57 (1H, s, H-4).

**^13^C-NMR** (700 MHz, CDCl_3_), δ_C_ 176.7 (C-5), 147.9 (C-4), 128.5 (C-2’), 127.1 (C-5’), 123.9 (C-4’), 114.3 (C-3’), 113.2 (C-3), 111.9 (C-1), 86.8 (C-2).

[M-H] is at m/e (237.9572), Calculated Mass (237.9580) Formula [M-H]^-^ (C_9_H_2_Cl_2_N_3_O)

**Data for compound 5**

IR spectrum for compound 5

 ^1^H-NMR spectrum of compound 5 (400 MHz, CDCl_3_, δ_TMS_= 0 ppm)

^13^C-NMR	spectrum of compound 5 (100 MHz, CDCl_3_, δ_TMS_= 0 ppm)

Table 2: ^13^C and ^1^H chemical shifts and of compound 5

| **N** | **δ_H_** | **δ_C_** | **N** | **δ_H_** | **δ_C_** |
| --- | --- | --- | --- | --- | --- |
| 1 | - | 111.9 | **1’** | 10.59 | - |
| 2 | - | 86.8 | **2’** | - | 128.5 |
| 3 |  | 113.2 | **3’** | - | 114.3 |
| 4 | 7.57 | 147.9 | **4’** | - | 123.9 |
| 5 | 9.71 (1H, s) | 176.7 | 5’ | - | 127.1 |

**Mass spectrum for compound 5**

## **Compound 6** (2,2'-((3,5-dichloro-1H-pyrrole-2,4-diyl)bis(methaneylylidene))dimalononitrile

**MP:** decomposed >250^0^C

**IR (ATR, Methanol):** 3255 cm⁻¹: (N-H stretching vibrations), 2221 cm⁻¹ (C≡N stretch), 1552 cm⁻¹ (N-H bending).

**^1^H-NMR** (700 MHz, CDCl_3_): δ_H_ 7.64 (s, 1H, H-4), 7.61 (s, 1H, H-4’).

**^13^C-NMR** (700 MHz, CDCl_3_): δ_C_ 150.4 (C-4’), 145.2 (C-4), 139.4 (C-5’’), 133.2 (C-2”), 126.3 (C-3’’), 117.4 (C-3’), 117.4 (C-4’’), 116.5 (C-1’), 115.6 (C-3), 115.0 (C-1), 77.9 (C-2), 71.0 (C-2’),

**MS:** [M-H] is at m/e (285.9700), Calculated Mass (285.9693) Formula [M-H]^-^ (C_12_H_2_Cl_2_N_5_)

**Data for compound 6**

IR spectrum of 6

^1^H-NMR spectrum of compound 6 (400 MHz, CH_3_OD, δ_TMS_= 0 ppm)

^13^C-NMR	spectrum of compound 6 (100 MHz, CDCl_3_, δ_TMS_= 0 ppm)

Table 3: ^13^C- and ^1^H chemical shifts and of compound 6

| **N^0^** | **δ_H_** | **δ_C_** | **N^0^** | **δ_H_** | **δ_C_** | **N^0^** | **δ_H_** | **δ_C_** |
| --- | --- | --- | --- | --- | --- | --- | --- | --- |
| 1 | - | 115.0 | **3’** | - | 117.4 | 5" | - | 139.4 |
| 2 | - | 77.9 | 4' | 7.61 (s, 1H) | 150.4 |  |  |  |
| 3 |  | 115.6 | 1" | - | - |  |  |  |
| 4 | **7.64** (s, 1H) | 145.2 | 2" | - | 133.2 |  |  |  |
| **1'** | - | 116.5 | 3'' | - | 126.3 |  |  |  |
| **2’** | - | 71.0 | 4'' | - | 117.4 |  |  |  |

**Mass spectrum for compound 6**

**Compound 8** (2,4-dichloro-5-((2,2-dimethyl-4,6-dioxo-1,3-dioxan-5-ylidene)methyl)-1H-pyrrole-3-carbaldehyde):

**MP:** Decomposed >250^0^C

**IR** (ATR, Methanol): 3358 (N-H stretching), 1729 cm⁻¹(C=O, ester carbonyl group), 1677 cm⁻¹ C=O, (aldehyde carbonyl group).

**^1^H-NMR** (700 MHz, CDCl3) δ_H_: 13.9 (1H, s, H-1’), 13.27 (1H, s, H-1), 9.99 (1H, s, CHO), 8.40 (1H, s. H-7), 1.78 (6H, s, H-8,and H-9).

**^13^C-NMR** (700 MHz, CDCl_3_) δ_C_: 182.2 (C-10), 164.5 (C-6), 162.5 (C-4), 137.9 (C-7), 131.3 (C-2’), 131.0 (C-5’), 124.4 (C-4’), 119.6 (C-3’), 105.4 (C-2), 105.4 (C-5), 27.5 (C-8 and C-9).

**MS:** [M-H]^-^ is at m/e (315.9789), Calculated Mass (315.9785 ) Formula [M-H]^-^ (C_12_H_2_Cl_2_N_5_)

**2,4-dichloro-5-((2,2-dimethyl-4,6-dioxo-1,3-dioxan-5-ylidene)methyl)-1H-pyrrole-3-carbaldehyde (8):**

- **IR (ATR, Methanol)**: 3358 cm^-1^ (N-H stretching), 3095 cm^-1^ (C_sp2_-H), 2924 and 2855 cm^-1^ (C-H, CHO), 1729 cm^-1^ (C=O, ester carbonyl group), 1677 cm^-1^ C=O, (aldehyde carbonyl group), 1552 cm^-1^(C=C) bonds in aromatic ring.
- **Mass (High resolution)**: in electrospray negative ionization mode the [M-H]^-^ is at m/e (315.9789), Calculated Mass (315.9785) Formula [M-H]^-^ (C_12_H_2_Cl_2_N_5_)
- **^1^H-NMR (700 MHz, CDCl3)**: δ_H_: 13.27 (1H, s, H-1), 8.40 (1H, s. H-7), 1.78 (6H, s, H-8 and H-9), 9.99 (1H, s, CHO), 13.9 (1H, s, H-1’).
- **^13^C-NMR (700 MHz, CDCl3):** δ_H_ 182.2 (C-10), 164.5 (C-6), 162.5 (C-4), 137.9 (C-7), 131.3 (C-2’), 131.0 (C-5’),.124.4 (C-4’), 105.4 (C-2), 105.4 (C-5), 119.6 (C-3’), 27.5 (C-8 and C-9).

**Data for compound 8:**

**Crystal data and structure refinement for 8**

An overlay of the two crystallographically independent molecules in the asymmetric unit of the crystal structure of **8**.

**Compound 9** (3,5-dichloro-4-((2,2-dimethyl-4,6-dioxo-1,3-dioxan-5-ylidene)methyl)-1H-pyrrole-2-carbaldehyde)

**MP:** Decomposed >250^0^C

**IR** (ATR, Methanol): 3280 cm⁻¹ (N-H), 1665 cm⁻¹ (C=O, carbonyl group).

**^1^H-NMR** (700 MHz, CDCl_3_) δ_H_: 13.3 (1H, s, H-1’), 9.66 (1H, s, H-10, CHO), 7.64 (1H, s. H-7), 1.68 (6H, s, H-8,and H- 9),

**^13^C-NMR** (700 MHz, CDCl3) δ_C_: 176.7 (C-10), 161.7 (C-4), 159.6 (C-6), 143.2 (C-7), 134.9 (C-2’), 128.2 (C-5’), 117.0 (C-3’), 115.8 (C-4’), 105.5 (C-2), 105.6 (C-5), 27.7 (C-8 and C-9).

**MS:** [M-H] is at m/e (315.9792), Calculated Mass (315.9785) Formula [M-H]^-^ (C_12_H_8_Cl_2_N_5_)

**Data for compound 9**

IR spectrum of 9

^1^H-NMR spectrum of compound 9 (175 MHz, CDCl_3_, δ_TMS_= 0 ppm)

^13^C-NMR spectrum of compound 9 (100 MHz, CDCl_3_, δ_TMS_= 0 ppm)

Table 4: (^13^C, ^1^H)-NMR Chemical shifts of compound 9 (400 MHz, CDCl_3_, δ_TMS_= 0 ppm)

| N^0^ | δ_H_ | δ_C_ | N^0^ | δ_H_ | δ_C_ |
| --- | --- | --- | --- | --- | --- |
| 1 | - | - | 7 | 7.64 | 143.2 |
| 2 | - | 105.5 | 1' | 13.3 | - |
| 3 | - | - | 2' | - | 134.9 |
| **4** | - | 161.7 | 3' | - | 117.0 |
| **5** | - | 105.6 | 4' | - | 115.8 |
| **6** | - | 159.6 | 5' | - | 128.2 |
| **8, 9** | 1.68 (6H, s) | 27.7 | 10 | 9.66 (1H, s) | 176.7.2 |

**Compound 10** (5,5'-((3,5-dichloro-1H-pyrrole-2,4-diyl)bis(methaneylylidene))bis(2,2-dimethyl-1,3-dioxane-4,6-dione):

**MP:** Decomposed > 250^0^C

**IR:** 3321 cm⁻¹ (N-H), 1731 and 1635 cm^-1^ (C=O, ester carbonyl groups).

**^1^H-NMR** (700 MHz, CDCl_3_) δ_H_: 13.9 (1H, s, H-1’), 8.40 (1H, s. H-7), 1.55 (12 H, s, H-8, H-9 and H- 8’ and H-9’).

**^13^C NMR**

**MS:** [M-H] is at m/e (237.9572), Calculated Mass (237.9580) Formula [M-H]^-^ (C_12_H_8_Cl_2_NO_5_).

**Data for compound 10**

**IR spectrum of 10**

**^13^C-NMR spectrum of compound 10 (100 MHz, CDCl_3_, δ_TMS_= 0 ppm)**

Table 5: (^13^C, ^1^H)-NMR Chemical shifts of compound 10 (400 MHz, CDCl_3_, δ_TMS_= 0 ppm)

| N^0^ | δ_H_ | N^0^ | δ_H_ | N^0^ | δ_H_ |
| --- | --- | --- | --- | --- | --- |
| 1 | - | 7 | 8.4 | 7' | 8.5 |
| 2 | - | 1' | 13.9 | 1’' | 13.3 |
| 3 | - | 2' | - | 2’' | - |
| **4** | - | 3' | - | 3’' | - |
| **5** | - | 4' | - | 4’' | - |
| **6** | - | 5' | - | 5’' | - |
| **8, 9** | 1.55 (6H, s) | - | - | - | - |

**Structure of 10**

**Mass spectrum for compound 10**

**Data of synthesized compounds:**

All the compounds decomposed at >250^0^C.

**2-((3,5-dichloro-4-formyl-1H-pyrrol-2-yl) methylene) malononitrile (4):**

- **Mass (High resolution):** electrospray negative ionization mode, the [M-H] is at m/e (237.9581), Calculated Mass (237.9580) Formula [M-H]^-^ (C_9_H_2_Cl_2_N_3_O)
- **IR (ATR**): 3442 cm^-1^ (N-H), 2924.7 cm^-1^ and 2853.17 cm^-1^ (C-H, CHO- stretching), 1645 cm^-1^ (C=O), 2219 (C≡N stretch), 1572.9 cm^-1^ (C=C stretching in aromatic rings),
- **^1^H-NMR** (700 MHz, CDCl_3_) δ_H_: 7.69 (1H, S, H-1), 9.98 (1H, s, H-5).
- **^13^C-NMR** (700 MHz, CDCl_3_) δ**_C_**: 181.6 (C-5), 140.5 (C-3), 131.8 (C-5’), 127.8 (C-3’), 122.9 (C-2’), 118.8 (C-4’) 114.5 (C-3), 112 (C-1), 75.1 (C-2).

**2-((3,5-dichloro-4-formyl-1H-pyrrol-2-yl)methylene)malononitrile (5):**

- **Mass (High resolution):** electrospray negative ionization mode the [M-H] is at m/e (237.9572), Calculated Mass (237.9580) Formula [M-H]^-^ (C_9_H_2_Cl_2_N_3_O)
- **IR (ATR, Methanol):** 3302.1 cm^-1^ (N-H stretch), 3030 (C_cp2_ -H), 2922 cm^-1^ and 2853 cm^-1^ C-H (CHO) stretching vibrations, 2221 cm^-1^ (C≡N stretch), 1647 cm^-1^ (C=O aldehydes stretching), 1574.4 cm^-1^ (C=C stretch, aromatic ring).
- **^1^H-NMR** (700 MHz, CDCl3): δ_H_ 10.59 (1H, bd-1’), 9.71 (1H, s, H-5), 7.57 (1H, s, H-4).
- **^13^C-NMR** (700 MHz, CDCl3), δ_C_ 176.7 (C-5), 147.9 (C-4), 128.5 (C-2’), 127.1 (C-5’), 123.9 (C-4’), 114.3 (C-3’), 113.2 (C-3), 111.9 (C-1), 86.8 (C-2).

**2,2'-((3,5-dichloro-1H-pyrrole-2,4-diyl)bis(methaneylylidene))dimalononitrile (6)**

- **MP:** decomposed >250^0^C
- IR (ATR, Methanol): 3255 cm^-1^ (N-H stretching vibrations), 2925 cm^-1^(C-H stretching sp2), 2221 cm^-1^ (C≡N stretch), 1552 cm^-1^ (N-H bending), 1553 cm^-1^ (C=C stretching in aromatic rings).
- Mass (High resolution) in electrospray negative ionization mode the [M-H] is at m/e (285.9700), Calculated Mass (285.9693) Formula [M-H]^-^ (C_12_H_2_Cl_2_N_5_)
- ^1^H-NMR (700 MHz, CDCl3): δ_H_ 7.64 (s, 1H, H-4), 7.61 (s, 1H, H-4’).
- ^13^C-NMR (700 MHz, CDCl3): δ_H_ 150.4 (C-4’), 145.2 (C-4), 139.4 (C-5’’), 133.2 (C-2”), 126.3 (C-3’’), 115.6 (C-3), 115.0 (C-1), 117.4 (C-3’), 117.4 (C-4’’), 116.5 (C-1’), 77.9 (C-2), 71.0 (C-2’).

**2,4-dichloro-5-((2,2-dimethyl-4,6-dioxo-1,3-dioxan-5-ylidene)methyl)-1H-pyrrole-3-carbaldehyde (8):**

- IR (ATR, Methanol): 3358 cm^-1^ (N-H stretching), 3095 cm^-1^ (C_sp2_-H), 2924 and 2855 cm^-1^ (C-H, CHO), 1729 cm^-1^ (C=O, ester carbonyl group), 1677 cm^-1^ C=O, (aldehyde carbonyl group), 1552 cm^-1^(C=C) bonds in aromatic ring.
- Mass (High resolution) in electrospray negative ionization mode the [M-H]^-^ is at m/e (315.9789), Calculated Mass (315.9785) Formula [M-H]^-^ (C_12_H_2_Cl_2_N_5_)
- ^1^H-NMR (700 MHz, CDCl3): δ_H_: 13.27 (1H, s, H-1), 8.40 (1H, s. H-7), 1.78 (6H, s, H-8,and H-9), 9.99 (1H, s, CHO), 13.9 (1H, s, H-1’).
- ^13^C-NMR (700 MHz, CDCl3): δ_H_ 182.2 (C-10), 164.5 (C-6), 162.5 (C-4), 137.9 (C-7), 131.3 (C-2’), 131.0 (C-5’),.124.4 (C-4’), 105.4 (C-2), 105.4 (C-5), 119.6 (C-3’), 27.5 (C-8 and C-9).

**3,5-dichloro-4-((2,2-dimethyl-4,6-dioxo-1,3-dioxan-5-ylidene)methyl)-1H-pyrrole-2-carbaldehyde (9)**

- IR (ATR, Methanol): 3280 cm^-1^ (N-H), 3080 cm^-1^ (C_sp2_-H), 2976-2881 cm^-1^ (C-H of CHO), 1665 cm^-1^ (C=O, carbonyl group).
- Mass (High resolution): in electrospray negative ionization mode the [M-H] is at m/e (315.9792), Calculated Mass (315.9785) Formula [M-H]^-^ (C_12_H_8_Cl_2_N_5_)
- ^1^H-NMR (700 MHz, CDCl3): δ_H_ 7.64 (1H, s. H-7), 1.68 (6H, s, H-8,and H- 9), 9.66 (1H, s, H-10, CHO), 13.3 (1H, s, H-1’).
- ^13^C-NMR (700 MHz, CDCl3) δ_H_: 176.7 (C-10), 161.7 (C-4), 159.6 (C-6), 143.2 (C-7), 134.9 (C-2’), 128.2 (C-5’), 105.6 (C-5), 105.5 (C-2), 117.0 (C-3’), 115.8 (C-4’), 27.7 (C-8 and C-9).

**5,5'-((3,5-dichloro-1H-pyrrole-2,4-diyl)bis(methaneylylidene))bis(2,2-dimethyl-1,3-dioxane-4,6-dione) (10)**

- **IR (ATR)**: 3321 cm⁻¹ (N-H), 2926 cm^-1^ (C_sp2_-H), 2855 cm-1 (C_sp3_-H), 1731 and 1635 cm^-1^ (C=O, ester carbonyl group).
- **Mass (High resolution)**: in electrospray negative ionization mode, the [M-H] is at m/e (237.9572), Calculated Mass (237.9580) Formula [M-H]^-^ (C_12_H_8_Cl_2_NO_5_).
- **^1^H-NMR (700 MHz, CDCl3)**: δ_H_ 13.9 (1H, s, H-1’), 8.40 (1H, s. H-7), 1.55 (12 H, s, H-8, H-9 and H- 8’ and H-9’),
